# Improving the aseptic transfer procedures in hospital pharmacies part C: evaluation and redesign of the transfer process

**DOI:** 10.1136/ejhpharm-2019-002034

**Published:** 2019-10-29

**Authors:** Frits A Boom, Paul P H Le Brun, Stefan Boehringer, Jos G W Kosterink, Daan J Touw

**Affiliations:** 1 Zaans Medical Center, Zaandam, The Netherlands; 2 Department of Clinical Pharmacy and Toxicology, Leiden University Medical Center, Leiden, The Netherlands; 3 Medical Statistics and Bioinformatics, Leiden University Medical Center, Leiden, The Netherlands; 4 Department of Clinical Pharmacy and Pharmacology, University Medical Center Groningen, Groningen, The Netherlands

**Keywords:** aseptic handling, aseptic transfer process, particle emission, sterile medical device, surface bioburden

## Abstract

**Objectives:**

To transfer sterile medical devices (SMD), infusion bags (IB), ampoules (A), injection vials (V) and infusion bottles (B) into a laminar airflow cabinet (LAF) or safety cabinet (SC) with a surface bioburden as low as possible.

**Methods:**

Surface bioburden of the outer layer of SMD, IB, A, V and B was determined by contact plates. Surface bioburden determination of critical spots on A, V and B (ampoule necks and stoppers) was determined by high-recovery swabs and contact plates. Particle emission from white cardboard boxes was determined by a particle counter.

**Results:**

The chances of a contaminated outer layer of SMD is negligible as long as they stay in their original boxes. The outer layer of double-packed IB can contain a considerable number of micro-organisms. As found in previous studies, the surface bioburden of A, V and B is low as long as they stay in their original cardboard boxes. Particle emission from white boxes is low. The necessity of a final disinfection step inside LAF/SC of critical sspots of A, V and B cannot be proven. SmallSMD, ampoules and injection vials can be transferred into the background areain their original white boxes. Other materials have to be unpacked in front ofthe lock while the operator wear disposable gloves. Disinfection of the outerlayer of IB, before transfer trough the lock, is advised. Tohave materials with a low chance of contamination in LAF/SC, transfer bypresentation for SMD and IB and using a sterile tray for disinfected materialsis an effective procedure. Wiping of ampoule necks and stoppers inside LAF/SC isadvised based on risk assessment.

Small SMD, ampoules and injection vials can be transferred into the background areain their original white boxes. Other materials have to be unpacked in front ofthe lock while the operator wear disposable gloves. Disinfection of the outerlayer of IB, before transfer trough the lock, is advised.

**Conclusion:**

When SMD, ampoules, injection vials and infusion bottles stay in their original boxes as long as possible, the aseptic transfer and the disinfection procedure can be maintained effectively and efficiently.

## Introduction

During aseptic handling many materials are used. These can be divided into materials with a sterile surface and materials with a non-sterile surface. Materials with a sterile surface are sterile medical devices (SMD) and double-packed infusion bags (IB). Materials with a non-sterile surface are glass and plastic ampoules, and injection vials and infusion bottles, all usually packed in cardboard boxes.

Materials are transferred in two steps into the working area (laminar airflow cabinet (LAF), safety cabinet (SC) or isolator (I)). The first step is the transfer through a lock from an adjacent area into the background area (the room in which the LAF/SC/I is housed: at least EU GMP Annex 1 grade D^1 2^). The materials can be stored there or used immediately. The second step is the transfer from the background area into LAF/SC/I.

Transfer is a critical process. If executed without enough precautions, micro-organisms can be dragged with the materials into LAF/SC/I and may contaminate the working space, the operator’s hands and eventually the products during aseptic handling.

SMD are wrapped and sterilised in a layer consisting of paper, plastic or a combination of both. The need for disinfection of this outer layer is doubtful if we keep in mind that SMD are sterilised in closed cardboard boxes. As long as these boxes are not opened, the chance of a contaminated outer layer of SMD will be negligible. This fact, in combination with a transfer process in which additional contamination will be kept low, is an opportunity to get wrapped SMD with a low bioburden into the background area and, next, to transfer the unwrapped SMD without any outside contamination into LAF/SC/I.

Infusion bags are wrapped and sterilised in a plastic layer and packed in cardboard boxes. Because of the lack of information about the outer layer surface bioburden we examined it.

Materials with a non-sterile surface have to be disinfected. The result of this process depends on the disinfectant, the disinfection method and the surface bioburden.^3^ We demonstrated that the surface bioburden of ampoules and vials before disinfection is low as long as they stay in their original boxes.^3 4^ Therefore, to prevent recontamination, these materials ideally have to be transferred into the background area in their original boxes. Boxes, however, are made out of cardboard, which can release viable and non-viable particles. Whether or not this is a real problem is unknown. Therefore, we determined the particle release from cardboard boxes and discuss its relevance on the viable and non-viable particle burden in the background environment.

After transfer into LAF/SC/I, vial stoppers are punctured by a needle or spike and therefore will have direct contact with the sterile solution. The same is true for ampoule necks because, during drawing up, contact between the syringe needle and the ampoule neck is almost inevitable. Therefore, additional disinfection of stoppers and ampoule necks inside LF/SC/I is general practice in hospital pharmacies in The Netherlands. We examined the efficacy of this additional disinfection.

Based on the results of this study, as well as those of parts A and B of this series of articles, we propose a transfer process for transferring materials inside LAF or SC with a low risk of dragging micro-organisms.^3 4^ The transfer into I is comparable to the transfer into LAF and SC, but there is little experience with I in The Netherlands. Therefore, we restrict our recommendations to LAF and SC.

## Materials and Methods

### Surface bioburden on the outer layer of wrapped SMD

In nine hospital pharmacies, samples from steel needles (Braun Sterican Mix 18 G x 1.2, 40 mm), plastic needles (Codan filter straws) and syringes (Becton and Dickinson, 5, 10 and 20 mL) were taken aseptically from the location in the background area where they were stored. Samples were transferred into LAF or SC and the paper surfaces were monitored by contact plates (Tryticase Soya Agar 55 mm diameter, Biotrading Benelux, The Netherlands). Contact time was 10 s. Steel needles were examined in sets of five and plastic needles and syringes were examined separately. For the number of samples, see table 1.

**Table 1:** Surface bioburden expressed as mean cfu per about 25 cm^2^ of the outer layer of wrapped syringes, steel needles (SN) and plastic needles (PN) in nine hospital pharmacies

Hospital	Syringes (n=10)			Steel needles (SN) / Plastic needles (PN) (n=10)	
	Volume (ml)	Mean cfu	SD	Mean cfu	SD
1	20	3.3	5.65	–	
2	10	0	0	0.9 (SN)	1,37
3	10	0	0	–	
4	10	0.1	0.32	0 (SN)	0
5	5	0.4	0.52	–	
6	5	0.8	1.73	0 (SN)	0
7	10	0.6	1.58	–	
8	10	0	0	0 (PN)	0
9	10	0.2	0.63	0 (PN)	0

n = number of samples examined; steel needles: 10 x a set of five needles were tested.

To get the paper surface as flat as possible, fingers were held on the back of this surface (see online supplementary figure 1). To improve contact, the plates were turned a little from left to right several times with light pressure. Only one 55 mm-diameter contact plate was used for each sample, which meant that up to a maximum of 23.7 cm^2^ of the whole surface was examined.

10.1136/ejhpharm-2019-002034.supp1 Supplementary data

After sampling, the contact plates were incubated for 7 days at 30+/-1°C and cfu were counted after 3 and 7 days.

### Surface bioburden on the outer layer of double-packed infusion bags

In the background area of different hospital pharmacies, samples were taken aseptically out of their original boxes:

- In three hospital pharmacies, 10 samples of 100 mL NaCl 0.9% IB (1 x Baxter, 2 x Kabi Fresenius)
- In four hospital pharmacies, 10 samples of 500 mL NaCl 0.9% IB (1 x Baxter, 3 x Kabi Fresenius)
- In five hospital pharmacies, 10 samples of 2000 mL parenteral nutrition (PN) bags (2 x Olimel N7E Baxter, 3 x Smofkabiven Kabi Fresenius)

The surface bioburdens were determined by contact plates (Tryticase Soya Agar 55 mm-diameter, Biotrading Benelux). Contact time was 10 s. To improve contact, the plates were turned a little from left to right several times with light pressure. Only one 55 mm-diameter contact plate was used for each sample, which meant that up to a maximum of 23.7 cm^2^ of the whole surface was examined.

After sampling, the contact plates were incubated for 7 days at 30+/-1°C and cfu were counted after 3 and 7 days.

### Surface bioburden on ampoules and vials before disinfection

In the background area in 10 hospital pharmacies, 10 samples of four different kinds of ampoules and vials were taken aseptically, just before disinfection. The sampled products were: 10 mL plastic ampoules (10 x Addamel Kabi Fresenius), 10 mL glass ampoules (7 x Vitintra Adult, 3 x Vitintra Infant Kabi Fresenius), 10 mL injection vials (7 x Soluvit N Kabi Fresenius, 3 x Cernevite Baxter) and 100 mL infusion bottles (7 x Water for injection Kabi Fresenius, 3 x Water for injection Braun).

Samples were transferred into LAF or SC and monitored by contact plates as described in part A.^4^ For infusion bottles, only one 55 mm-diameter contact plate was used, which meant that about 15% of the surface of a 100 mL vial was examined.^4^

After sampling, the contact plates were incubated for 7 days at 30+/-1°C and cfu were counted after 3 and after 7 days.

### Particle emission from white cardboard boxes

The experiments were executed in a SC. The original white cardboard boxes with Soluvit N, Vitintra adult 10 mL and Supleven 10 mL (all Fresenius-Kabi), and empty white carboard boxes used in the pharmacy for packaging ampoules and vials, were rubbed together continuously. Particles were counted five times during 4 min with a Met One HHPC 2+handheld airborne particle counter (flowrate 0.0028 m^3^ air per minute). The distance between the probe of the particle counters and the rubbed boxes was 10 cm.

### Determination of the bioburden on stoppers and ampoule necks

The experiments were executed in a LAF cabinet.

Glass ampoules (Vitintra adult 10 mL, Fresnius-Kabi) and injection vials (Soluvit N, Fresenius-Kabi) were taken straight from their original boxes and placed in a LAF cabinet. Plastic flip-off caps were removed from the vials. Two sampling methods were used:

1. Swab: 20 ampoule necks and 20 vial stoppers from non-disinfected and disinfected* glass ampoules were thoroughly wiped by a moistened high- recovery nylon-flocked swab (Quantiswabs bioMerieux) and directly streaked on a TSA plate (Tryticase Soya Agar 90 mm-diameter, Biotrading Benelux).
2. Contact plate: 35 non-disinfected and 35 disinfected* vial tops (aluminium crimp cap and rubber stopper) were pressed with light pressure and with a holding time of 10 s on TSA plates (Tryticase Soya Agar 90 mm diameter, Biotrading Benelux).
  *Disinfection according to the one-step two-towel disinfection method as described in part B.^3^

After sampling, the TSA plates were incubated for 7 days at 30+/-1°C and cfu were counted after 3 and after 7 days.

## Results

### Surface bioburden on the outer layer of wrapped SMD

Table 1 shows the surface bioburden on about 25 cm^2^ of the outer layer of wrapped SMD. To be able to assess the distribution of cfu over the different samples, SD are given.

### Surface bioburden on the outer layer of double-packed infusion bags

Table 2 shows the surface bioburden on about 25 cm^2^ of the outer layer of double-packed IB. Because of the great variety in cfu counts on the 119 IB (between 0 and more than 50 cfu per investigated surface), the results are subdivided into different groups (0 cfu, 1–5 cfu and so on). High cfu counts were not correlated to a particular manufacturer, kind of bag or volume.

**Table 2:** Surface bioburden expressed as mean cfu per about 25 cm^2^ of the outer layer of three kinds of infusion bags

Number of cfu	Number of bags		
	100 mL	500 mL	PN
0	7	18	36
1–5	13	12	10
6–10	5	5	0
10–50	4	3	1
>50	1	2	2
Total number of bags tested	30	40	49

First horizontal row: we found zero cfu on 7 100 mL bags, 18 500 mL bags and 36 PN bags: second horizontal row we found 1–5 cfu on 13 100 mL bags, 12 500 mL bags and 10 TPN bags etc.

### Surface bioburden on ampoules and vials before disinfection

**Figure 1:**
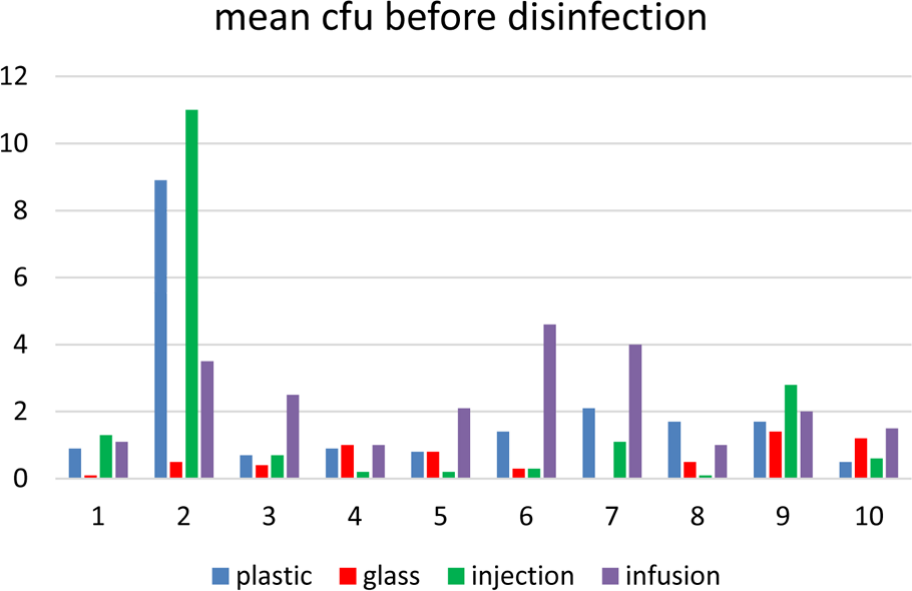
Surface bioburden expressed as mean* cfu on plastic and glass ampoules and on injection vials and infusion bottles in 10 hospital pharmacies before disinfection. *Mean out of 10 samples. Horizontal axis: 1, 2, 3 etc. are hospital 1, 2, 3 etc. Vertical axis: mean cfu/sample. Note: For infusion bottles only about 15% of the vial surface was examined.^3^

Figure 1 shows the surface bioburden on ampoules and vials before disinfection in 10 hospital pharmacies. For infusion bottles, only about 15% of the vial surface was examined.^4^

Hospitals 3 and 5 had the overall best results (mean cfu 0.6 of all ampoules and vials) and Hospital 2 had the worst results (mean cfu 6.8 of all ampoules and vials).

### Particle emission from white cardboard boxes

The mean number of particles from each kind of cardboard box after five-times rubbing are expressed in table 3 as particle per m^3^. The maximum limits for airborne particles in Grade C in operation are also presented in table 3.^2^

**Table 3:** Particle emission after rubbing white cardboard boxes expressed as mean particles ≥0.5 and ≥5 µm per m^3^

Cardboard boxes from	Particles ≥0.5 µm/m^3^ (n=5)		Particles ≥5 µm/m^3^ (n=5)	
	Mean	SD	Mean	SD
Soluvit N*	81 959	69 022	8049	7181
Vitintra adult*	22 260	7404	3513	1561
Ampoules 10 mL	75 146	37 863	16 064	13 237
Supleven	34 086	10 635	3460	688
Overall mean†	53 362	19 946	7771	3788
Limits airborne particle for Grade C in operation	3 520 000	–	29 000	–
P-value for overall mean; limit is reference group	<0.001		<0.001	

*Laminated cardboard.

†Overall mean of all 20 determinations.

n, number of samples examined.

### Determination of the bioburden on stoppers and ampoule necks

The results of the bioburden determination on the vial stoppers and the ampoule necks are summarised in table 4. It shows that even before disinfection, most of the samples contain no cfu.

**Table 4:** Surface bioburden, expressed as number of samples with growth (pos), on ampoule necks and vial stoppers before and after one-step two-towel disinfection

	Quantiswab				TSA plate			
	Non-disinfected (n=20)		Disinfected (n=20)		Non-disinfected (n=35)		Disinfected (n=35)	
	pos	SD*	pos	SD*	pos	SD*	pos	SD*
Vial stopper	1 (2 cfu)	0.24	0	0	1 (1 cfu)	0.17	0	0
Ampoule neck	2 (both 1 cfu)	0.32	1 (1 cfu)	0.24	–	–	–	–

*SD is based on dichotomised values (zero cfu against one or more cfu).

n, number of samples examined; pos, number of samples with one or more cfu (between brackets = number of cfu).

## Discussion

For designing an effective and efficient transfer process we needed information about the surface bioburden of different kinds of materials with and without a sterile surface, stored in different places. A lot of information is already available in parts A and B.^3 4^ With the additional information, described in this article, we were able to redesign the transfer process.

The results in tables 1 and 2 provide information about only a part of the surface bioburden of the outer layer of the examined SMD and IB (23.7 cm^2^ at a maximum). It should not be interpreted as information on the whole surface bioburden but can be helpful for the redesign of the transfer process.

Because of the low number of samples and the differences in the transfer processes in the participating hospital pharmacies, the results in table 1 and in figure 1 cannot be used for comparing bioburdens and/or transfer processes of SMDs, ampoules and vials.^4^ However, the results show that without precautions, the bioburden can increase during transfer and storage.

### Transfer of materials into the background environment

The risk of contamination via the airborne route is low because aseptic handling is done using closed systems. Different studies have confirmed this.^5–7^ Therefore, the recommended background area in The Netherlands is Grade D.^8^ In other countries Grade C or even Grade B is recommended.^1^ To be clear, the recommendations of the transfer process described below do not apply for a Grade B background.

#### Materials with a sterile surface

As mentioned in the Introduction, the chance of a contaminated outer layer of SMD will be negligible as long as they stay in their original boxes. After opening, contamination will occur as shown in table 1. Poor storage conditions as well as manipulating with ungloved hands will further increase the level of contamination. In Hospital 1, for example, SMD are held with ungloved hands outside the background area and stored inside the background area in open bins, that are not always completely empty before being refilled. In contrast, we found low bioburdens when gloves were used in combination with storage in closed cupboards (Hospital 8 for example). This leads us to advise the following procedure: always wear non-sterile or sterile gloves, irrespective of the place where SMD are taken from, unpack the original boxes in front of the materials lock, carry over the SMD into empty bins or trays, transfer these trays through the lock and store them in closed cupboards. Injection needles, as well as other small SMD, can be stored like ampoules and vials (see below, Materials with a non-sterile surface) in their original white cardboard boxes in the background area.

Infusion bags are also sterilised in a second outer layer (see Introduction). Compared with the surface bioburdens of non-disinfected ampoules and vials (figure 1), the outer layer of double-packed IB can sometimes contain a considerable number of cfu (table 2). Also, these outer layers are not always clean, especially in the case of the PN bags. Therefore, it is better to unpack the IB in front of the lock, clean and disinfect the outer layer using alcohol-impregnated wipes, and then put the bags directly into the lock. Obviously, all these activities have to be done with (non-)sterile gloved hands.

### Materials with a non-sterile surface

As mentioned previously, it is important to have materials with a low-surface bioburden before disinfection.^3^ As shown in figure 1, these bioburdens are generally low. The relatively high bioburdens found in Hospital 2 (figure 1) are caused by storage in open boxes on open shelves and handling the materials without gloves.

In Hospital pharmacies 3, 4 and 5 ampoules and injection vials are stored in their original white boxes up to use. The surface bioburdens are low and are comparable with the bioburdens in materials stored under the same conditions, as described earlier.^3 4^ These results confirm the assumption that ampoules and injection vials ideally should be transferred into the background area in their original cardboard boxes. An additional advantage of this way of working is a lower workload compared with the transfer of single ampoules and vials.

Cardboard, however, is not recommended in the background area because of particle release. Our experiments on particle emission contradict this recommendation (see table 3). From the four kinds of boxes the overall mean number of particles, after rubbing, were significantly below the limits for airborne particles in Grade C in operation. Moreover, the experiments simulated a worst-case situation. Boxes are not normally rubbed together. Particle emission from white cardboard boxes will therefore have no measurable influence on the particle burden in the background area.

Theoretically, particle emission from white laminated cardboard should be lower than from white non-laminated cardboard. Our results, however, did not confirm this expectation (see table 3).

Cardboard is a well-known source of viable particles, among which are spore-forming bacteria.^9^ The risk increases where cardboard becomes damp. However, the greatest source of viable particles in the background area are the operators.^10^ Compared with this source, the number of viable particles emitted from white cardboard boxes is negligible. All materials with a non-sterile surface will be disinfected before being transferred into LAF/SC (see below, Transfer of materials into LAF or SC). In part B we showed that spore-forming bacteria on these materials will disappear like other micro-organisms by wiping with well-impregnated alcoholic wipes.^3^ We therefore think that white cardboard inside the background area is not a risk for an increase in viable particles inside LAF/SC.

Like ampoules and vials, the surface bioburden of infusion bottles is low as long as they stay in their original boxes.^4^ However, infusion bottles are packed in brown cardboard boxes. This type of cardboard is less clean than white cardboard. Therefore, we advise taking infusion bottles out of their original boxes in front of the lock with gloved hands and putting them directly into the lock. For practical reasons, the transfer procedure for infusion bottles and IB can be harmonised. When doing so, infusion bottles must also be wiped.

**Figure 2:**
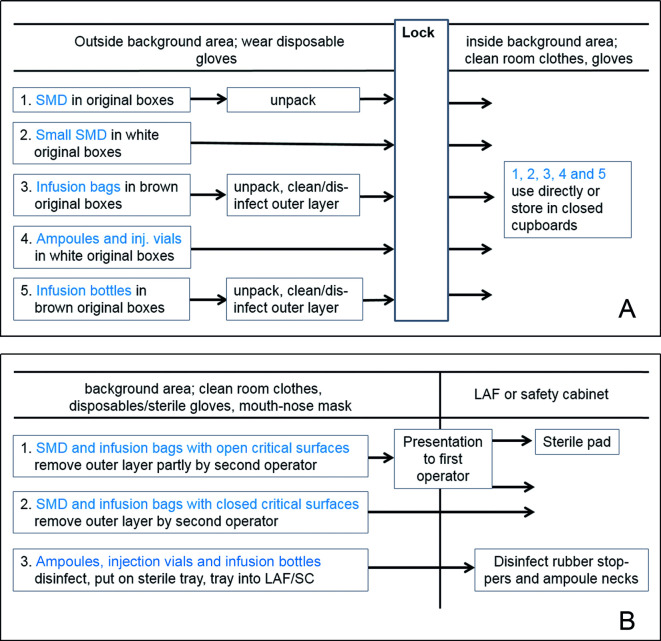
Transfer of materials with a sterile and a non-sterile surface. (A) Transfer into the background area. (B) Transfer from the background area into LAF/SC. Note to (A): transfer of IB and infusion bottles can be harmonised to one procedure (see text).

The optimal transfer for materials with a sterile and a non-sterile surface into the background area is summarised in figure 2A. This transfer process not only guarantees low-surface bioburdens, it also simplifies the procedure. For example:

- Only a part of the material has to be unpacked before transfer.
- Disinfection can be restricted to IB (and infusion bottles in the case of harmonisation).
- Storage of ampoules and vials inside the background environment is easier to handle in boxes compared with single items.

### Transfer of materials into LAF or SC

Starting point for the procedure described below is working with two operators.^1 11^

#### Materials with a sterile surface

Before use, SMD have to be unwrapped. If unwrapped materials are brought into LAF/SC the risk of contact between critical spots (syringe tips, needles, openings of tubes and connection points) and the disinfected, but not sterile, surface of the worktop is relatively high. Therefore, we advise that the second operator partly unwraps SMD in front of LAF/SC. This operator presents the sterile side to the first operator (see online supplementary figure 2a) who pulls out the SMD completely and places it into LAF/SC on a sterile pad (see online supplementary figure 2b). For IB, we also advise a transfer by presentation. The critical spots of these bags, however, are protected. Therefore, it is not necessary to put them on a sterile pad (see online supplementary figure 2b). SMD, where the critical spots are protected by a cap, such as plastic spikes, can be unwrapped in front of LAF/SC and brought into LAF/SC by the second operator and put next to the sterile pad (see online supplementary figure 2b).

10.1136/ejhpharm-2019-002034.supp2 Supplementary data

### Materials with a non-sterile surface

As explained earlier, we advise keeping disinfected materials in a sterile tray on a sterile surface (see part B figure 2).^3^ The tray with the disinfected materials can be transferred into a LAF or SC and can later be used for collecting waste such as used vials, ampoules, needles and syringes inside the LAF/SC. This makes a separate waste box unnecessary.

Because of direct contact between needles and spikes and vial stoppers, as well as the high chance of touching the ampoule neck by a needle, a last disinfection step inside LAF or SC of vial stoppers and ampoule necks is general practice in The Netherlands. With the experiments to determine the bioburden on these critical spots we tried to find out the effectiveness of this additional disinfection step. Ampoule necks can be reached by swabs only, therefore we had to use this sampling method. As explained in part A, the recovery from traditional cotton, rayon or polyester swabs is low.^4^ Therefore, we used the high-recovery Quantiswab.^12^ The almost flat top of an injection vial (stopper and crimp cap) makes it possible to monitor these surfaces by contact plates. The recovery is comparable with the Quantiswab (40%–60%), but after sampling there is no need for additional laboratory work.^3^

Before disinfection the surface bioburdens of the critical spots are already low (in total 4 cfu on 75 samples, see table 4) and comparable with results on vial stoppers found by Cockcroft et al.^13^ After thoroughly wiping (two-towel technique, see^3^) the bioburden decreased to 1 cfu on 75 samples. To prove whether or not additional wiping is significantly better, a great number of samples is needed. For example: 1 cfu on 75 samples means 1.3% of the samples contaminated with 1 or more cfu. To prove that less than 1% of the samples is contaminated, one needs a sample size of over 500 (CI=95%).^14^ Because of the workload of this experiment and the decision, based on risk assessment, to continue with additional wiping, irrespective of the outcome of a second study, we decided not to perform this experiment.

In figure 2b, based on our results, the optimum process for transfer of materials into the LAF/SC is shown. The above-described transfer technique of SMD by presentation, as well as one-step disinfection (wiping) restricted to ampoules and vials only, will keep the transfer procedure simple, while low- surface bioburdens are still guaranteed.

## Conclusion

Small SMD, ampoules and injection vials can be transferred into the background area in their original white boxes. Other materials have to be unpacked in front of the lock while the operator wears disposable gloves. Disinfection of the outer layer of IB, before transfer through the lock, is advised.

To have materials with a low chance of contamination in LAF/SC, transfer by presentation for SMD and IB and using a sterile tray for disinfected materials is an effective procedure. Wiping of vial stoppers and ampoule necks inside LAF/SC is advised based on risk assessment.

**What this paper adds**

**What is already known on this subject**

- During aseptic handling, many materials (ampoules, injection vials, infusion bottles, infusion bags and sterile medical devices) are used.
- The transfer of these materials into a laminar airflow cabinet, safety cabinet or isolator is a critical process from a microbiological point of view.

**What this study adds**

- Ampoules, injection vials and infusion bottles have a low-surface bioburden as long as they stay in their original boxes.
- The chance of contaminated outer layers of sterile medical devices is negligible, as long as they stay in their original boxes.
- When sterile medical devices, ampoules, injection vials and infusion bottles stay in their original boxes as long as possible, the aseptic transfer procedure and the disinfection procedure can be maintained effectively and efficiently.

## Data Availability

Data are available upon reasonable request.
