# Supplementary material for: Improving the aseptic transfer procedures in hospital pharmacies part C: evaluation and redesign of the transfer process
Source: Eur J Hosp Pharm. 2019 Oct 29;29(1):12–7. doi: 10.1136/ejhpharm-2019-002034 (PMC8717784; doi:10.1136/ejhpharm-2019-002034)
Supplement: Supplementary data [file ejhpharm-2019-002034supp001.pdf]

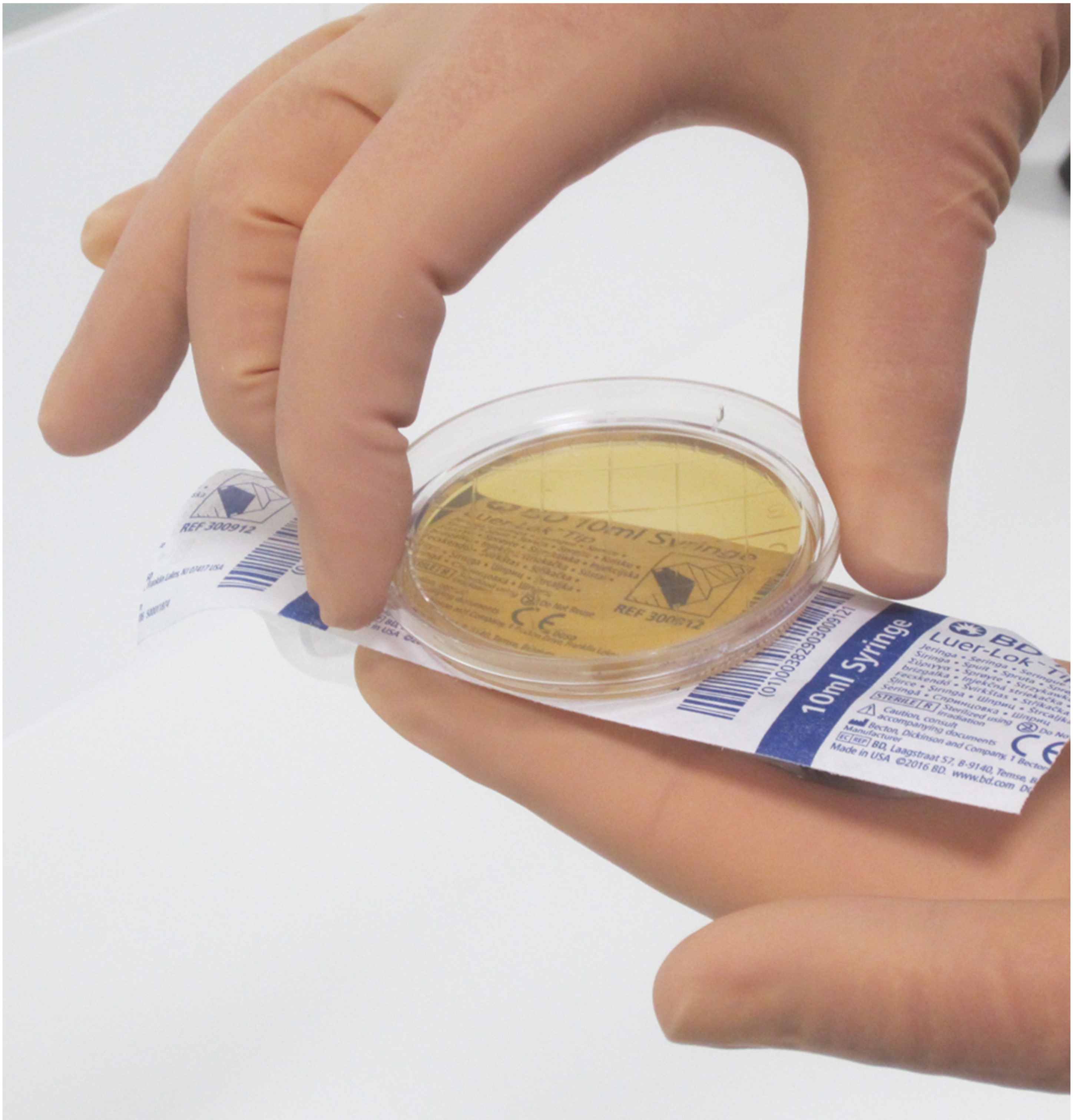

Supplementary figure 1 Surface bioburden determination of the wrapped outer layer of a syringe by a contact plate.
